# Analysis of the Compressive Behavior of Plywood Under Seawater and Cryogenic Temperature Effects

**DOI:** 10.3390/ma18081836

**Published:** 2025-04-16

**Authors:** Jong-Min Choi, Hee-Tae Kim, Tae-Wook Kim, Dong-Ha Lee, Jeong-Hyeon Kim, Jae-Myung Lee

**Affiliations:** 1 Department of Naval Architecture & Ocean Engineering, Pusan National University, Busan 46241, Republic of Korea; 2 Hydrogen Ship Technology Center, Pusan National University, Busan 46241, Republic of Korea

**Keywords:** plywood, seawater, immersion, compression strength, cryogenic, aging tests

## Abstract

The global demand for liquefied natural gas (LNG) has led to a significant increase in the number of LNG carriers (LNGCs), consequently elevating the risk of operational accidents. Unlike conventional vessels, LNGCs present a high risk of fire and explosion and involve extensive repair times and costs due to the complex structure of the cargo containment system (CCS). This study investigates the effects of seawater exposure on the uni-axial compressive properties of plywood used in LNGC CCS structures, with the goal of establishing material strength criteria that could reduce repair requirements. The analysis focuses on the NO96 CCS, which incorporates the highest volume of plywood among existing designs. In this configuration, compressive strength is a critical design parameter. Therefore, the mechanical response of plywood was evaluated under both room temperature and cryogenic conditions (−163 °C), simulating the LNG operating environment. The results demonstrate that plywood exhibited increased compressive strength after three hours of seawater and saltwater immersion, although the rate of improvement diminished with extended exposure. In contrast, specimens immersed in distilled water showed a consistent reduction in compressive strength. Furthermore, cryogenic temperatures significantly enhanced the compressive strength compared to ambient conditions. This study establishes a methodology for assessing the mechanical performance of plywood under marine and cryogenic conditions, contributing to its reliable application in LNG carrier structures.

## 1. Introduction

Wood is primarily composed of tubular fibers or cells, consisting mainly of cellulose, hemicelluloses, and lignin. Among these components, cellulose is the strongest polymer, offering high mechanical strength due to its linear orientation and high degree of polymerization [1]. Plywood is an engineered wood product manufactured by bonding together multiple thin veneers, with adjacent layers oriented at up to 90° angles to improve strength and stability. It is widely recognized as a structural material among wood-based composites due to its excellent dimensional stability, high strength-to-weight ratio, and strong resistance to chemical and impact damage [2]. The mechanical properties of plywood are influenced by several factors, including the quality of the veneer, layer configuration, type of adhesive, and bonding conditions [3]. Plywood is commonly used in applications, such as structural supports in construction, industrial paneling, vehicle interiors, and shipbuilding [4].

The strength of plywood is particularly dependent on parameters, such as wood species, density, grain orientation, veneer quality, number of layers, and adhesive resin type [5,6]. Given these favorable characteristics, plywood has seen increasing application in the maritime and offshore industries, particularly in membrane-type LNG cargo containment systems (CCSs) such as the GTT Mark III and NO96 systems. Plywood is one of the few wood-based materials capable of maintaining structural integrity under cryogenic conditions, including exposure to internal and external mechanical loads arising from LNG sloshing during transport in rough sea conditions or ballast operations [7]. However, exposure to harsh marine environments over time can degrade its shape, strength, and performance, necessitating strict quality control standards in shipping and offshore applications.

To ensure safety and reliability, materials used in marine environments must be evaluated for changes in physical and mechanical properties due to seawater exposure. For LNG carriers, in particular, internal structural materials must comply with regulations requiring resistance to LNG leakage for up to 15 days following an accident. Therefore, material performance should be assessed under simulated seawater exposure conditions. In the case of natural materials like plywood, prior research has typically focused on performance changes under either cryogenic conditions or seawater immersion independently. However, studies addressing the combined effects of both seawater exposure and cryogenic temperatures—especially in the context of internal structural materials used in LNG CCSs—remain scarce.

Several studies have examined the effects of seawater exposure on wood-based composites. Kolat et al. [8] investigated the fracture toughness of sandwich structures with various plywood core materials, estimating environmental degradation via seawater pre-conditioning. While the fracture toughness of sandwich systems with wood and plywood cores decreased, that of other systems increased under seawater exposure. Rangaraj et al. [9] explored the effects of marine environments on wood/thermoplastic composites, particularly focusing on fatigue behavior under combined moisture and mechanical loading. Najafi et al. [10] conducted flexural tests on wood–polypropylene composites and observed a correlation between flexural strength and water absorption. Although numerous studies have examined the effect of seawater on wood, the specific influence on plywood remains inadequately addressed. In general, composites immersed in seawater show greater weight gain and lower flexural strength compared to those immersed in distilled water.

In addition to moisture-related effects, several studies have evaluated the influence of cryogenic temperatures on plywood. Kim et al. [11] examined the mechanical properties and failure behavior of melamine–urea–formaldehyde (MUF) resin plywood under cryogenic conditions, concluding that MUF resin exhibited higher fracture strength than phenol–formaldehyde (PF) resin. From a mechanical performance perspective, MUF resin is considered a viable alternative to PF resin for LNG applications. Kim et al. [12] further studied the influence of cryogenic thermal loading on plywood, focusing on the effects of grain orientation, and found that MUF resin plywood retained mechanical performance comparable to PF resin under cryogenic conditions. LeVan et al. [13] evaluated fire-retardant-treated pine plywood subjected to cyclic thermal conditions simulating real-world temperature fluctuations. Choi et al. [14] investigated the effect of cryogenic temperatures on the buckling behavior of plywood reinforced with glass fiber/epoxy laminates. However, while these studies provide insights into the effects of low temperatures, they do not assess the combined impact of moisture and temperature, such as seawater immersion followed by cryogenic exposure.

This study aims to evaluate the compressive strength of plywood following seawater exposure, with the goal of establishing repair criteria for LNG CCS components affected by accidental seawater ingress. It is generally assumed that moisture exposure—particularly from seawater—negatively affects plywood strength. However, if strength degradation is negligible or even improved after drying, these findings may serve as a valuable basis for redefining material replacement criteria, potentially reducing maintenance costs and minimizing the environmental impact from plywood waste. For instance, the Gaztransport and Technigaz (GTT) guidelines recommend evaluating the strength degradation of internal CCS materials due to seawater exposure [15]. Accordingly, this study investigates the effects of aqueous contamination on the compressive strength of plywood, a material with a capillary-based moisture absorption structure, by considering practical conditions such as seawater leakage, alongside control groups immersed in distilled water and saltwater solutions.

## 2. Background and Problem Description

### 2.1. Background

**Figure 1:**
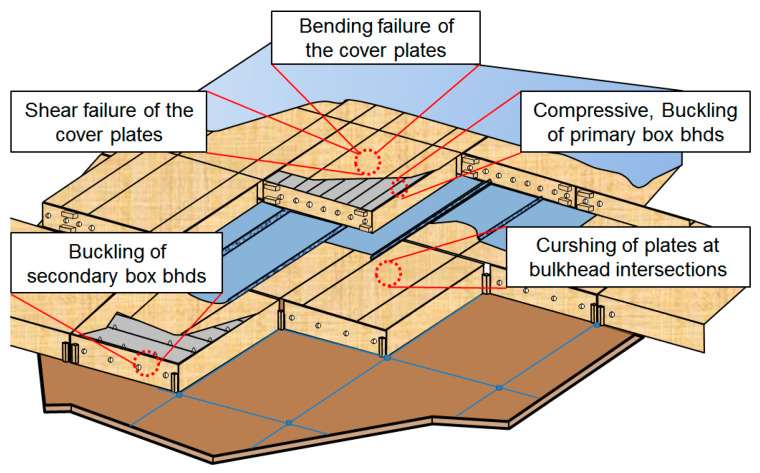
Failure modes considered for the NO96 system [16].

In liquefied natural gas (LNG) cargo containment systems (CCSs) commonly used in LNG carriers, various structural loads—including crushing, compression, and buckling—are exerted on the system and are significantly intensified by the sloshing of LNG during transit (Figure 1) [16]. Plywood serves as a critical structural component in CCSs, primarily as part of the insulation system and inner hull structure.

Several cases of CCS failure have been reported, including LNG leakage resulting from local deformation caused by sloshing, as summarized in Table 1. A recent example involves damage sustained by an LNG bunker vessel following a collision with a cargo ship [17]. When the insulation system is compromised, LNG can leak into the insulation layers. In more severe incidents, the leaked LNG may breach the secondary insulation barrier and reach the carrier’s inner hull plate. Persistent leakage into the inner hull can ultimately result in structural degradation.

In addition to sloshing-induced damage, impact events such as collisions and groundings must also be considered in CCS safety assessments [18]. Previous parametric studies have evaluated the thermal integrity of inner hull plates under LNG-filled conditions and during potential leakage events. Furthermore, failure mode and effect analysis (FMEA) has been employed to identify and assess various accident scenarios and their potential consequences, underscoring the need for systematic risk evaluation (Table 2).

### 2.2. Problem Definition

Among the various loads acting on CCSs, compressive load is one of the primary forces affecting structural elements (Figure 1). Therefore, it is crucial to enhance the structural integrity of CCS components against applied loads. Under ballast water immersion, plywood must maintain its compressive strength at cryogenic temperatures (−160 to −162 °C) to ensure LNG remains in its liquid state. According to Gaztransport and Technigaz (GTT) code requirements, core materials in cargo tanks, such as plywood and polyurethane foam, must withstand exposure to seawater [15]. In the MARK-III insulation system, polyurethane foam is susceptible to environmental factors such as methane gas exposure due to sealing failures in seawater environments. As a result, data on the mechanical property degradation of plywood under seawater exposure are essential, as it is the primary material used in the NO96 insulation system. Unlike the MARK-III system, where panel replacement is feasible since it is primarily handled by shipyards, the NO96 insulation system faces significant challenges. Most shipyards have ceased production of NO96 insulation due to supply chain disruptions, particularly the rising cost of nickel, which has made sourcing Invar, the barrier material, increasingly difficult. In this situation, if a flooding accident occurs in an NO96 insulation system, supplying additional insulation boxes for repairs becomes highly challenging. Therefore, urgent research is needed to provide evidence on the effects of seawater contamination on the NO96 insulation system. The most structurally vulnerable component of the NO96 insulation system is the web structure that reinforces the interior of the plywood box. Since plywood is the most susceptible material in the system, understanding its buckling strength under seawater immersion is critical. This study aims to evaluate the buckling strength degradation of plywood as a basis for future replacement criteria for the NO96 insulation system in the event of seawater exposure. The present study assumes the most severe conditions, where either the inner hull of the CCS or the insulation system is cracked and damaged. Under these extreme conditions, an evaluation of plywood’s compressive mechanical properties considering both seawater exposure and temperature effects is necessary.

## 3. Experiments

### 3.1. Materials

**Figure 2:**
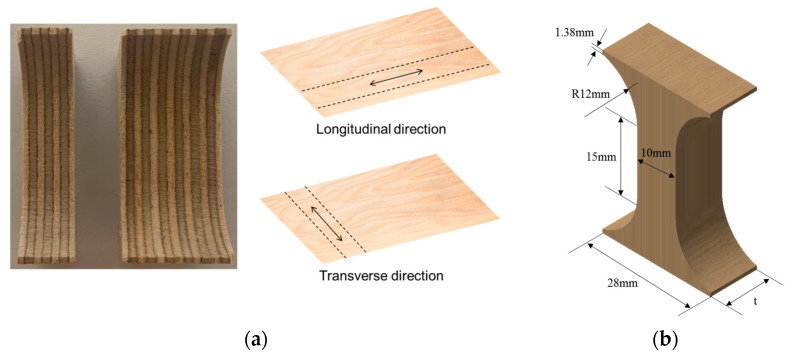
(**a**) The laminated plywood in the longitudinal and transverse directions and (**b**) the specimen dimension for the compression test.

Plywood consists of thin layers of wood laminated together to enhance mechanical strength and reduce shrinkage. In the shipbuilding and offshore industries, plywood with a thickness of 9 to 12 mm is commonly used as a core material [18]. In this study, compression tests were performed on plywood specimens of different thicknesses to investigate their transverse compressive behavior. The test specimens measured 28 mm × 40 mm × T mm (length × width × thickness), with multiple layers laminated in both longitudinal and transverse directions along the fiber orientation. Two thicknesses, 9T and 18T, were used, as shown in Figure 2a. Compression tests followed ASTM D695-23 to observe fine buckling behavior in individual fibers under compressive loading [19]. The testing procedure was performed according to Kopp’s method [20], with specimens designed to precisely measure compressive behavior during testing (Figure 2b) [21,22,23].

### 3.2. Compression Testing Apparatus and Method

Compression tests were performed using a universal testing machine (UTM, Kyoungsung Testing Machine Co., Ltd., Anyang-si, Korea) designed for composite materials, with a maximum load capacity of 50 kN. The maximum load and crosshead speed were set to 0.001 and 400 mm/min, respectively. A cryogenic chamber was employed to maintain either room temperature or cryogenic conditions during testing. To reach cryogenic temperatures, liquid nitrogen (LN_2_) was continuously injected into the chamber until the target temperature was achieved. A solenoid valve regulated the injection process, automatically stopping when the internal thermocouple detected the target temperature and resuming when the temperature rose above the set point [24].

**Figure 3:**
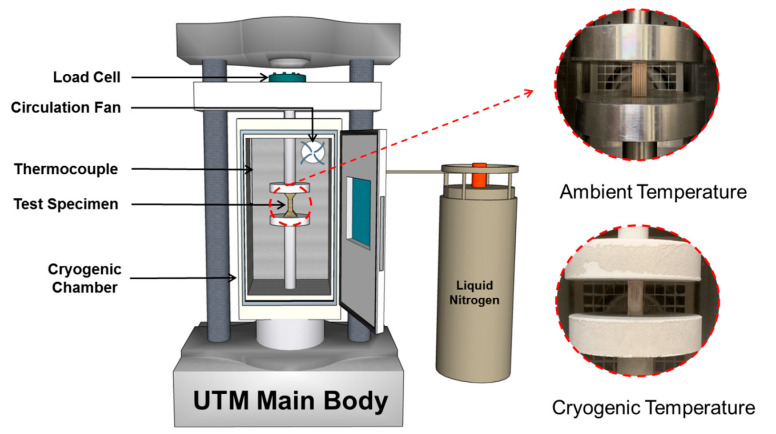
Schematic diagram and photograph of universal testing machine used to measure the mechanical characteristics of the plywood.

Figure 3 shows a photograph of the compression testing setup under cryogenic conditions. The compression jig was mounted to the UTM, and the plywood specimens were secured in position. All the tests were conducted at a constant strain rate of 0.166 mm/s.

### 3.3. Specimen for Immersion Condition

Nazmul Alam D.M. [25] reported that water absorption significantly affects the mechanical properties of plywood, particularly during the initial 24 h of immersion. Since the most pronounced changes typically occur in the early stages of exposure [15], immersion durations of 3, 6, and 12 h were selected for this study. To evaluate the effect of moisture and salinity, the test specimens were immersed in seawater, saltwater, and distilled water using specially designed plastic containers.

Following immersion, all the specimens were naturally dried for 24 h to eliminate internal moisture and ensure that the subsequent mechanical tests reflected the material’s inherent behavior. The natural drying step was introduced to simulate realistic post-contamination conditions and avoid humidity-induced bias in the results.

To isolate the influence of salinity, the specimens were first tested after immersion in distilled water. Then, to simulate seawater salinity, a 3.5% saltwater solution was prepared by dissolving 2.63 g/L NaCl, 0.427 g/L MgSO_4_, 0.2 g/L MgCl_2_, 0.088 g/L CaCl_2_, 0.056 g/L KCl, and 0.021 g/L NaHCO_3_ in distilled water. This composition reflects the ionic makeup of natural seawater and enables a controlled comparison across varying salinity conditions. To ensure statistical reliability, each test condition was repeated five times. A summary of the compression test conditions is provided in Table 3.

## 4. Results and Discussion

### 4.1. Effect of Room Temperature on Different Immersion Conditions

**Figure 4:**
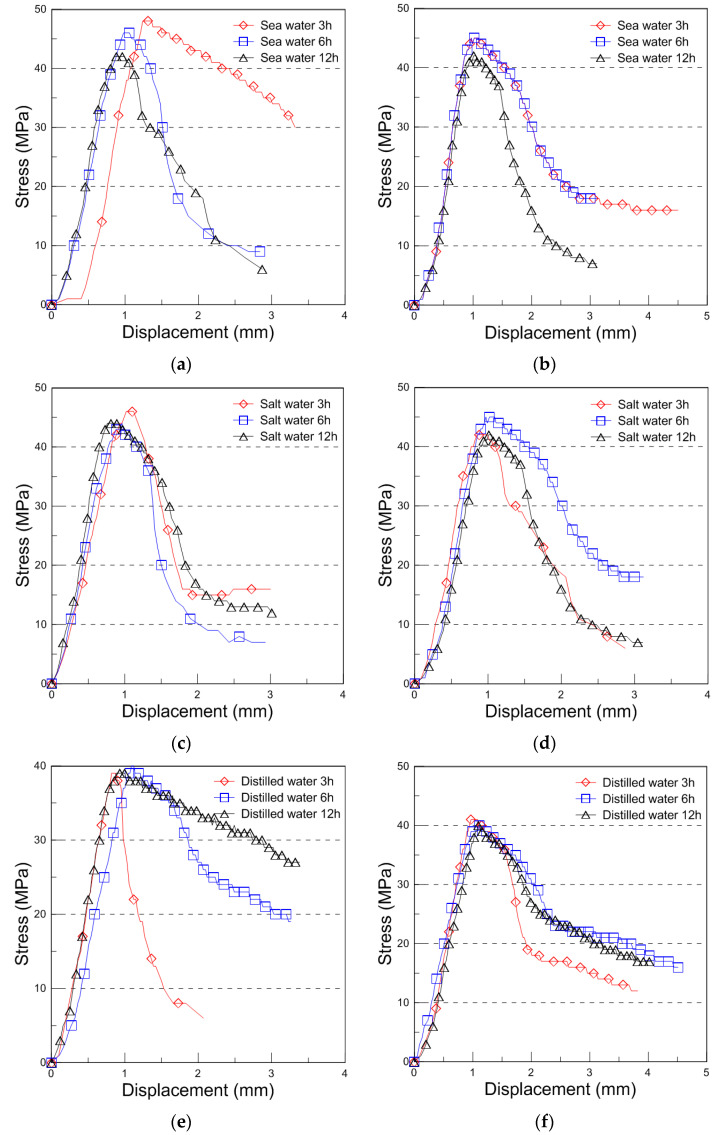
Compressive stress with displacement curve for the seawater, saltwater, and distilled water immersion ((**a**): 9 mm, (**b**): 18 mm thickness for the seawater immersion, (**c**): 9 mm, (**d**): 18 mm thickness for the saltwater immersion, (**e**): 9 mm, and (**f**): 18 mm thickness for the distilled water immersion).

**Figure 5:**
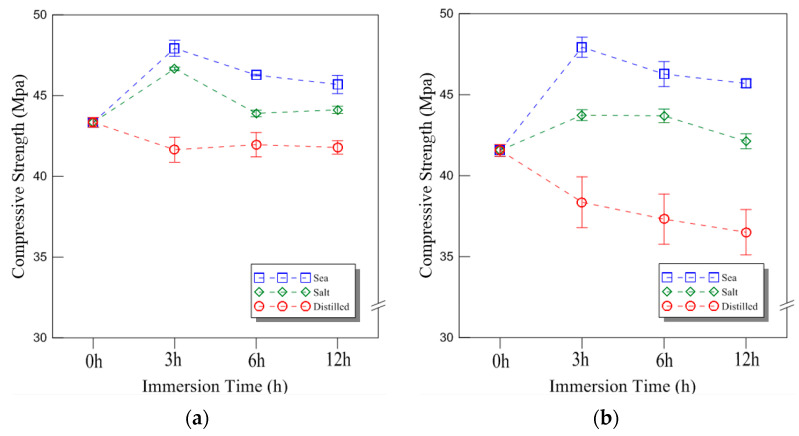
The compressive strength of the plywood for the different thicknesses under room temperature: (**a**) 9 mm; (**b**) 18 mm thickness.

The typical compressive behavior of plywood under seawater, saltwater, and distilled water immersion at room temperature is shown in Figure 4, illustrating the relationship between compressive stress and displacement. The comparative strength data for different immersion conditions and durations are presented in Figure 5, with the results analyzed for plywood specimens of 9 mm and 18 mm thickness. For the 9 mm plywood, the mean compressive strength values were 47.93 MPa, 46.27 MPa, and 45.69 MPa after 3, 6, and 12 h of seawater immersion, respectively. Similarly, for the 18 mm plywood, the corresponding values were 47.10 MPa, 45.90 MPa, and 43.06 MPa. These findings indicate that compressive strength increased by approximately 10% for the 9 mm plywood and 13% for the 18 mm plywood after 3 h of seawater immersion. This increase is primarily attributed to osmotic dehydration, which induces shear stress within the plywood layers. The moisture content of the plywood ranged from 6% to 14%, with a relative humidity of 45% at room temperature [16]. In the case of saltwater immersion, the compressive strength showed an increase after 3 h, with the 9 mm plywood gaining 8% in strength and the 18 mm plywood gaining 5%. However, as the immersion time extended to 6 and 12 h, the rate of increase declined, with the strength improvements reduced to 1% and 2% for the 9 mm plywood and 5% and 1% for the 18 mm plywood, respectively. The highest compressive strength was observed after 3 h of immersion, after which the increase became marginal. This trend aligns with the findings from seawater immersion, though the effects were slightly less pronounced. Beyond 12 h, the compressive strength showed minimal further increase, indicating that prolonged exposure to saltwater had little additional impact. For distilled water immersion, the compressive strength exhibited a distinct declining trend. After 3 h, the 9 mm plywood experienced a 4% decrease, while the 18 mm plywood showed an 8% decrease in strength. The reduction became more significant with longer immersion times, with the 9 mm plywood losing 3% and 4% after 6 and 12 h, respectively, while the 18 mm plywood showed a 10% and 12% decrease over the same periods. The substantial loss in strength is attributed to increased moisture absorption, which weakens the material’s structure by reducing its anisotropic Young’s modulus. This phenomenon aligns with findings reported by Ozyhar et al. [26].

Overall, the results indicate that seawater and saltwater immersion initially enhance plywood’s compressive strength, particularly after 3 h of exposure, while longer immersion times lead to diminishing returns. In contrast, distilled water immersion consistently weakens plywood due to increased moisture absorption, which degrades its mechanical properties. These findings highlight the importance of considering immersion conditions when evaluating plywood’s performance in marine environments.

**Figure 6:**
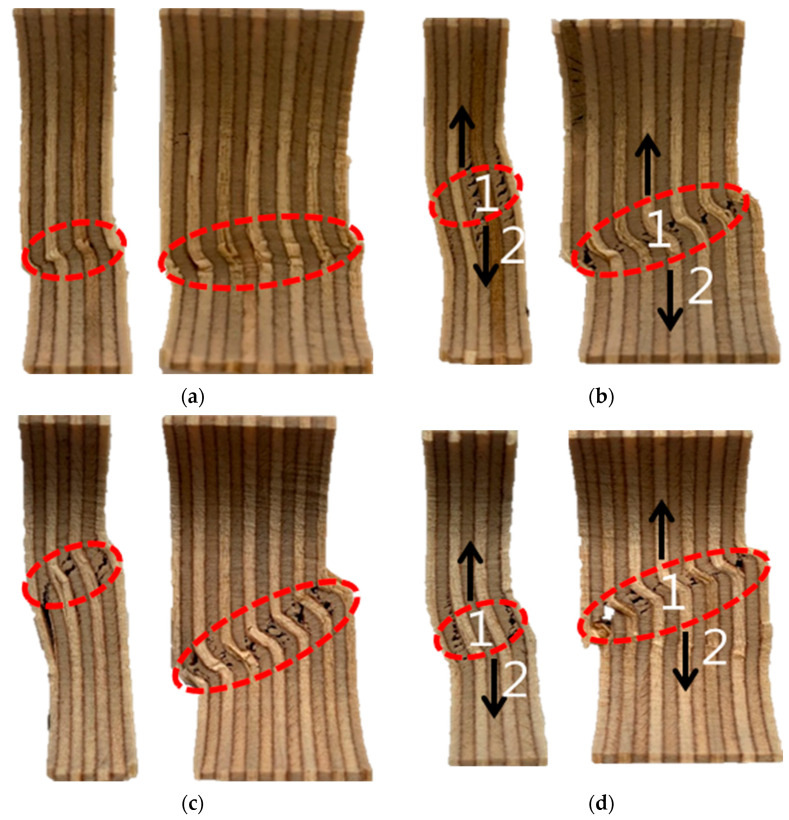
The microscopic image of the fractured specimens for the seawater immersion plywood of 9 and 18 mm thickness for (**a**) 3 h and (**b**) 12 h and the saltwater immersion plywood of 9 and 18 mm thickness for (**c**) 3 h and (**d**) 12 h. 1. Innermost veneers break; 2. Fractures propagate along the grain.

Figure 6 shows microscopic images of fractured 9 mm and 18 mm plywood specimens after immersion in seawater, saltwater, and distilled water. The specimens exhibited distinct deformation characteristics at room temperature, depending on the type of immersion. The red-highlighted areas in Figure 6 indicate the fracture surfaces of the tested plywood specimens and the regions where buckling occurred. Fractures in the veneer primarily originated from the innermost layers, with cracks propagating along the grain. When the plywood was immersed in seawater for 3 h and subjected to compressive loading, microcracks began to form within the plywood. These cracks, oriented perpendicular to the loading direction, spread from the inner veneer toward the outer veneer. Comparing the results after 3 and 12 h of immersion, a reduction in strength was observed, attributed to the increased effects of salt crystallization. Table 4 shows the detailed compressive strength obtained from the test results under room temperature.

### 4.2. Effect of Cryogenic Temperature on Different Immersion Conditions

**Figure 7:**
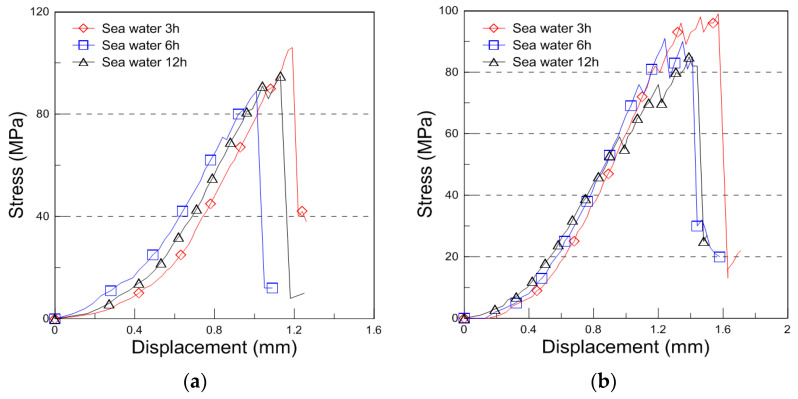

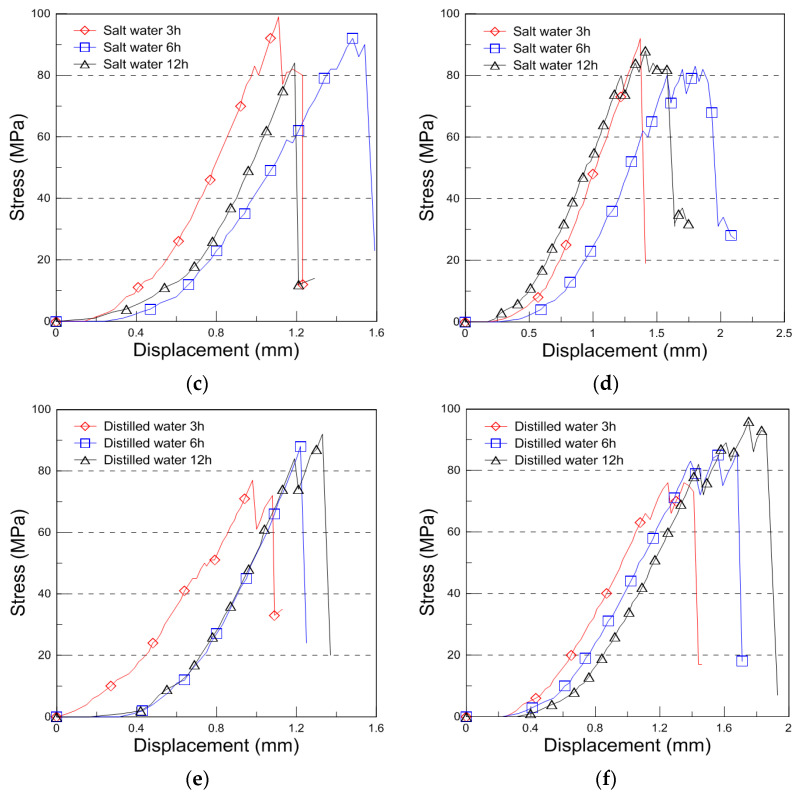
Compressive stress with displacement curve for the seawater, saltwater, and distilled water immersion under cryogenic condition ((**a**): 9 mm, (**b**): 18 mm thickness for the seawater immersion, (**c**): 9 mm, (**d**): 18 mm thickness for the saltwater immersion, (**e**): 9 mm, and (**f**): 18 mm thickness for the distilled water immersion).

The compressive behavior of the plywood at −163 °C under different immersion conditions (seawater, saltwater, and distilled water) is shown in Figure 7, illustrating the compressive stress–displacement curves. At cryogenic temperatures, the failure behavior became more complex, resulting in jagged load–displacement curves due to brittle failure mechanisms.

**Figure 8:**
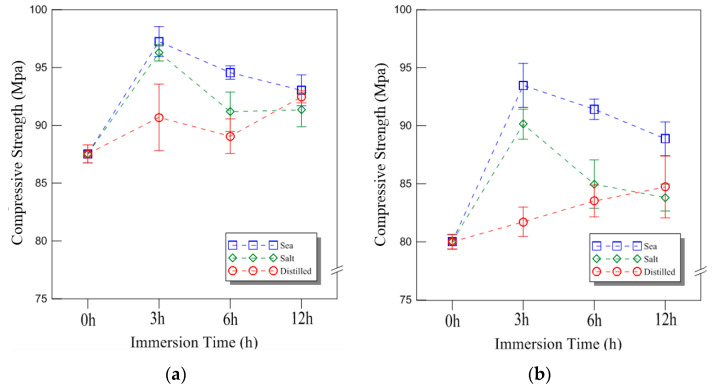
The compressive strength of the plywood for the different thicknesses under cryogenic temperature: (**a**) 9 mm; (**b**) 18 mm thickness.

The compressive strength of the 9 mm plywood under cryogenic conditions is shown in Figure 8a. The strength values for seawater immersion increased by 11%, 8%, and 6% for 3, 6, and 12 h, respectively. For saltwater immersion, the increase was 10%, 4%, and 4%, while for distilled water immersion, the values increased by 4%, 2%, and 6%, respectively. Compared to the room temperature conditions, the plywood exhibited significantly higher compressive strength at cryogenic temperatures, particularly after 3 h of immersion, where the increase was most pronounced. These results indicate that plywood maintains superior compressive strength under cryogenic conditions, making it a viable material for LNG containment applications. The compressive strength results for the 18 mm plywood are shown in Figure 8b. After 3, 6, and 12 h of seawater immersion, the compressive strength increased by 17%, 14%, and 11%, respectively. For saltwater immersion, the increase was 13%, 6%, and 5%, while for distilled water immersion, the values increased by 2%, 4%, and 6%, respectively. The seawater and saltwater immersion conditions resulted in relatively higher compressive strength at 18 mm thickness. Interestingly, distilled water immersion also showed an increase in compressive strength over time, which was inconsistent with previous findings for seawater and saltwater immersion at room temperature. The overall trend at cryogenic temperatures revealed higher compressive strength values and a greater rate of increase compared to room temperature conditions. This behavior can be attributed to the environment-dependent properties of plywood. The effect of moisture content in the plywood also played a role in the compressive strength trend, particularly for distilled water immersion under cryogenic conditions. The plywood used in this study contained approximately 6% to 14% moisture, and as the environmental temperature decreased, frozen moisture influenced the mechanical behavior. Consequently, a continuously increasing trend in compressive strength was observed, which differed from the decreasing trends seen under room temperature conditions. These phenomena were further confirmed through fracture analysis.

**Figure 9:**
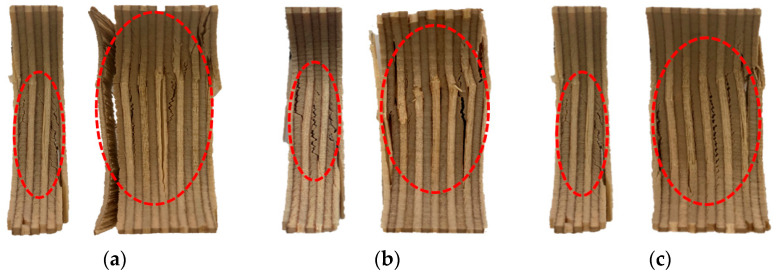
The microscopic image of the fractured specimens of the 9 and 18 mm plywood for (**a**) the seawater, (**b**) saltwater, and (**c**) distilled water immersion.

Figure 9 presents microscopic images of the fractured 9 mm and 18 mm plywood specimens after immersion in seawater, saltwater, and distilled water. As shown, the specimens exhibited distinct deformation characteristics under cryogenic conditions depending on the immersion type. The red-highlighted areas in Figure 9 indicate the fracture surfaces of the tested plywood specimens. The veneers primarily exhibited interlaminar shear failure, particularly within the weaker middle layers, accompanied by delamination at the interfaces and localized compressive failure. These failure modes differed significantly from those observed at room temperature, where buckling was the dominant failure mechanism. These findings further confirm that plywood demonstrates enhanced compressive strength under cryogenic conditions compared to room temperature. Table 5 shows the detailed compressive strength obtained from the test results.

From these findings, it can be concluded that plywood immersed in seawater or saltwater for 3 h exhibited a maximum increase in compressive strength of 10% at room temperature and 17% at cryogenic temperatures, regardless of plywood thickness. However, as immersion time increased to 12 h, the rate of strength increase declined for both seawater and saltwater conditions. In contrast, distilled water immersion led to an 8–10% decrease in compressive strength after 3 h, with a continuous decline observed up to 12 h. Based on these trends, plywood subjected to compressive loads remains structurally reliable after 3 h of seawater or saltwater immersion, regardless of temperature conditions. The behavior of freshwater-immersed plywood at cryogenic temperatures differed from that at room temperature, likely due to differences in the surface tension between freshwater and saltwater. Previous studies indicate that freshwater evaporates faster than saltwater, but in the case of wood, moisture diffusion follows capillary action, which is primarily governed by fluid surface tension. Since saltwater has a higher surface tension than freshwater, moisture diffuses more rapidly in a saltwater environment. Under controlled drying conditions (24 h), internal moisture likely diffused faster in saltwater-exposed plywood, whereas freshwater-exposed plywood retained more internal moisture.

As a result, ice crystal formation within freshwater-exposed plywood may have provided additional structural support at cryogenic temperatures, enhancing its mechanical properties. This difference in moisture diffusion and ice formation provides a possible explanation for the unexpected increase in compressive strength under cryogenic conditions for plywood immersed in distilled water.

## 5. Conclusions

This study investigated the compressive strength of plywood subjected to seawater, saltwater, and distilled water immersion under both room temperature and cryogenic conditions. The key findings are summarized as follows:

- Seawater immersion resulted in a 10–13% increase in compressive strength after 3 h, with the rate of increase diminishing over time, likely due to osmotic dehydration-induced shear stress.
- Saltwater immersion produced a moderate strength increase of approximately 10% after 3 h, whereas distilled water immersion led to an 8–10% decrease in strength over the same period.
- Under cryogenic conditions, plywood immersed in seawater and saltwater for 3 h exhibited a strength increase exceeding 10%; however, the strength gradually declined with extended immersion time.
- Cryogenic temperatures significantly enhanced compressive strength, particularly after short-term exposure (3 h) to seawater and saltwater, indicating temperature-dependent changes in mechanical properties.
- Distilled water immersion under cryogenic conditions resulted in a consistent increase in compressive strength, potentially due to moisture freezing within the structure.
- A microscopic analysis revealed distinct fracture characteristics: weaker veneer-adhesive bonding in saltwater-immersed specimens and buckling failure in those immersed in distilled water.
- Moisture diffusion rates varied across immersion types, with saltwater promoting faster diffusion than freshwater, influencing the drying behavior and internal stress distribution of plywood.

These results highlight the significant influence of immersion conditions and temperature on the mechanical performance of plywood. The findings contribute valuable insights for evaluating material durability in LNG cargo containment systems and optimizing repair strategies following seawater contamination.

## Figures and Tables

**Table 1:** Incidents of LNG CCS.

No.	Ship/Facility Name	Damage	LNG Spill	Comment
1	(‘66) Methane Progress	No	Yes	∙ Cargo leakage reported
2	(‘70) Arctic Tokyo	Yes	No	∙ Sloshing in No.1 LNG tank broke cable tray supports, perforating the barrier and leaking LNG into the inter-barrier
3	(‘78) Khannur	Yes	No	∙ Collision with cargo ship Hong Hwa
4	(‘85) Ramdane Abane	Yes	No	∙ Collision while loaded; port bow affected
5	(‘90) Bachir Chihani	Yes	No	∙ Sustained structural cracks allegedly caused by stressing and fatigue in inner hull
6	(‘93) Indonesian LNG Facility	No	No	∙ LNG leaked from an open line during pipe modification, entered a storm sewer, expanded, and ruptured the pipes
7	(‘13) Kisarazu Ko Offshore Beacon	Yes	No	∙ Vessel A had portside dents and cracks, while Vessel B sustained bow crush damage and bulbous bow dents

**Table 2:** FMEA study of barrier damage.

Process	System Failure	Cause	Effect
Panel installation	∙ Panel capsizing ∙ Clash with forklift ∙ Jig and tool falling	∙ Binding imperfection ∙ Careless driving ∙ Handle without care	∙ Member damage ∙ Replacement of broken panel
NH3 test for LNG cargo	∙ Remaining inspection material	∙ Unskilled workman	∙ Inspection error
	∙ Paraphernalia falling	∙ Management without care	∙ Falling items broken ∙ Replacement of broken panel
Membrane installation	∙ Fault construction by dust	∙ Incomplete dust removal from cutting works	∙ Seam error ∙ Hole re-formation
	∙ Jig and clamp falling ∙ Fire by electric leakage ∙ Carriage falling	∙ Unskilled workman ∙ Electric leakage ∙ Abandoned broken gear	∙ Delay of schedule ∙ Replacement of broken panel
	∙ Moving membrane falling ∙ Member falling when pump tower turnover	∙ Binding imperfection	∙ Replacement of broken panel
	∙ Member falls during installation and removal	∙ Absence of bottomplywood	∙ Careless forklift driving ∙ Scaffolding collapse ∙ Arbitrary dismantling ∙ Replacement of broken panels

**Table 3:** Test scenarios of plywood for compressed.

Number	Specimen Type	Temperature	Thickness (mm)	Immersion (h)
1	Untreated	Room temperature	9	3 6 12
2	Seawater			
3	Saltwater	Cryogenic	18	
4	Distilled water			

**Table 4:** The detailed compressive strength obtained from the test results under room temperature.

Type	Thickness (mm)	Immersion (h)	Compressive Strength (MPa)
Untreated	9	0	43.5 ± 0.4
	18	0	41.7 ± 0.4
Seawater	9	3	47.9 ± 0.5
		6	46.2 ± 0.2
		12	45.6 ± 0.6
	18	3	47.1 ± 0.7
		6	45.9 ± 0.7
		12	43.1 ± 0.3
Saltwater	9	3	46.6 ± 0.1
		6	43.9 ± 0.2
		12	44.1 ± 0.3
	18	3	43.8 ± 0.3
		6	43.8 ± 0.4
		12	42.2 ± 0.4
Distilled water	9	3	41.7 ± 0.8
		6	42.0 ± 0.8
		12	41.9 ± 0.4
	18	3	38.4 ± 1.6
		6	37.4 ± 1.6
		12	36.5 ± 1.5

**Table 5:** The detailed compressive strength obtained from the test results under cryogenic temperature.

Type	Thickness (mm)	Immersion (h)	Compressive Strength (MPa)
Untreated	9	0	87.5 ± 0.8
	18	0	80.0 ± 0.6
Seawater	9	3	97.3 ± 1.3
		6	94.6 ± 0.5
		12	93.1 ± 1.3
	18	3	93.5 ± 2.0
		6	91.6 ± 0.9
		12	88.9 ± 1.3
Saltwater	9	3	96.3 ± 0.5
		6	91.1 ± 1.8
		12	91.4 ± 1.5
	18	3	90.1 ± 1.3
		6	84.9 ± 2.1
		12	83.8 ± 1.2
Distilled water	9	3	90.6 ± 2.8
		6	89.0 ± 1.5
		12	92.5 ± 0.5
	18	3	81.7 ± 1.3
		6	83.5 ± 1.5
		12	84.8 ± 2.3

## Data Availability

The original contributions presented in this study are included in this article. Further inquiries can be directed to the corresponding authors.
